# *Spirulina platensis* Ameliorates Oxidative Stress Associated with Antiretroviral Drugs in HepG2 Cells

**DOI:** 10.3390/plants11223143

**Published:** 2022-11-17

**Authors:** Thabani Sibiya, Terisha Ghazi, Jivanka Mohan, Savania Nagiah, Anil A. Chuturgoon

**Affiliations:** 1 Discipline of Medical Biochemistry and Chemical Pathology, School of Laboratory Medicine and Medical Sciences, College of Health Sciences, Howard College Campus, University of KwaZulu-Natal, Durban 4013, South Africa; 2 Medical Programme, Department of Human Biology, Faculty of Health Sciences, Nelson Mandela University Missionvale, Port Elizabeth 6059, South Africa

**Keywords:** highly active antiretroviral therapy (HAART), *Spirulina platensis*, oxidative stress, reactive oxygen species, antioxidant, HAART toxicity

## Abstract

Lately, *Spirulina platensis* (SP), as an antioxidant, has exhibited high potency in the treatment of oxidative stress, diabetes, immune disorder, inflammatory stress, and bacterial and viral-related diseases. This study investigated the possible protective role of *Spirulina platensis* against ARV-induced oxidative stress in HepG2 cells. Human liver (HepG2) cells were treated with ARVs ((Lamivudine (3TC): 1.51 µg/mL, tenofovir disoproxil fumarate (TDF): 0.3 µg/mL and Emtricitabine (FTC): 1.8 µg/mL)) for 96 h and thereafter treated with 1.5 µg/mL *Spirulina platensis* for 24 h. After the treatments, the gene and protein expressions of the antioxidant response pathway were determined using a quantitative polymerase chain reaction (qPCR) and Western blots. The results show that *Spirulina platensis* decreased the gene expressions of *Akt* (*p* < 0.0001) and *eNOS* (↓*p* < 0.0001) while, on the contrary, it increased the transcript levels of *NRF-2* (↑*p* = 0.0021), *Keap1* (↑*p* = 0.0002), *CAT* (↑*p* < 0.0001), and *NQO-1* (↑*p* = 0.1432) in the HepG2 cells. Furthermore, the results show that *Spirulina platensis* also decreased the protein expressions of NRF-2 (↓*p* = 0.1226) and pNRF-2 (↓*p* = 0.0203). Interestingly, HAART-SP induced an NRF-2 pathway response through upregulating NRF-2 (except for FTC-SP) (↑*p* < 0.0001), *CAT* (↑*p* < 0.0001), and *NQO-1* (except for FTC-SP) (↑*p* < 0.0001) mRNA expression. In addition, NRF-2 (↑*p* = 0.0085) and pNRF-2 (↑*p* < 0.0001) protein expression was upregulated in the HepG2 cells post-exposure to HAART-SP. The results, therefore, allude to the fact that *Spirulina platensis* has the potential to mitigate HAART-adverse drug reactions (HAART toxicity) through the activation of antioxidant response in HepG2 cells. We hereby recommend further studies on *Spirulina platensis* and HAART synergy.

## 1. Introduction

Due to high infection and fatality rates, the human immunodeficiency virus (HIV) remains a global health problem [1]. In 2021, around 8.2 million people in South Africa were living with HIV [2]. Prior to the year 2021, an estimated 1.5 million additional people became newly infected with HIV globally, with 680,000 AIDS-related deaths [1,2,3,4]. The availability of antiretrovirals (ARVs) has helped those living with HIV/AIDS live longer. In 2020, around 27.5 million HIV-infected people worldwide had access to ARVs, while roughly 5.6 million HIV-infected South Africans had access [1,2,5].

Highly active antiretroviral therapy (HAART) combines many types of antiretroviral drugs to lower the viral load of an HIV-positive person [6,7,8]. HAART has several negative side effects that lead to metabolic syndrome, which includes oxidative stress and inflammation, despite its success in extending the lives of HIV-infected patients. The most popular nucleoside/nucleotide reverse-transcriptase inhibitors (NRTIs) are 2′,3′-dideoxy-5fluoro-3′-thiacytidine (FTC), (-)-L-2′,3′-dideoxy-3′-thiacytidine (3TC), and tenofovir disoproxil fumarate (TDF), which form part of the first-line therapy [9,10,11,12,13]. Moreover, 3TC, FTC, and TDF are recommended for pre-exposure prophylaxis (PrEP) by the World Health Organization (WHO) [14,15,16,17,18,19]. Additionally, 3TC is part of post- exposure prophylaxis (PEP) [20]. It is imperative to explore solutions to the negative side effects associated with these ARV drugs. 3TC, an important component of HAART, which can also be used to prevent HIV infection after potential exposure, has been linked to increased ROS, endothelial cell injury, and cardiovascular complications in HIV-infected individuals [20]. Another HAART backbone component, TDF, is a prodrug of tenofovir that is used to treat HIV infection and is associated with mitochondrial alterations and oxidative stress [21]. FTC, an analogue of 3TC, is used to inhibit the replication of HIV-1 and HIV-2. However, FTC is linked to the development of lipodystrophy syndrome, which is a result of low mitochondrial toxicity [22]. Supplementation with an antioxidant agent such as *Spirulina platensis*, which has been shown to protect against oxidative stress [23,24,25], can help to minimize the oxidative stress during HAART treatment. 

*Spirulina platensis* is a blue-green microalga that Mexican and African people eat as a traditional cuisine [26,27]. *Spirulina platensis* can be found in volcanic lakes’ alkaline water [23,24,25]. *Spirulina platensis* has also been shown to reduce oxidative stress [23,26,28,29] and increase mitochondrial health [30,31,32,33]. *Spirulina platensis* has been discovered to have a variety of therapeutic characteristics in addition to its well-known nutritional value. The health benefits found in *Spirulina platensis* include the inhibition of viruses, such as HIV-1 [34,35], and the inhibition of metabolic diseases [23,28]. *Spirulina* has also been credited with cancer- and viral infection-suppressing abilities [36]. When ingested, it can strengthen the immune system’s humoral and cellular systems [27]. *Spirulina platensis* has been associated with hypoglycaemia [37], hypolipidemic [38], and antihypertensive [39] effects. Furthermore, antioxidant complexes such as phycocyanin and phycocyanobilin are found in *Spirulina platensis* [23,24,25]. Moreover, it also contains high amounts of carotenoids and phenolic compounds [40]. Studies have identified that the bioactive compounds found in *Spirulina platensis* include carotenoids, phenols, chlorophylls, phycocyanin, polyunsaturated fatty acids (PUFAs), glycosides, flavonoids, and alkaloids [41,42,43,44,45]. *Spirulina platensis* can also help to prevent the onset of atherosclerosis [28] and diabetes [23]. In view of the above information, the aim of this study was to investigate the possible protective role of *Spirulina platensis* against HAART-induced oxidative stress in HepG2 cells. 

## 2. Results

### 2.1. Akt

**Figure 1:**
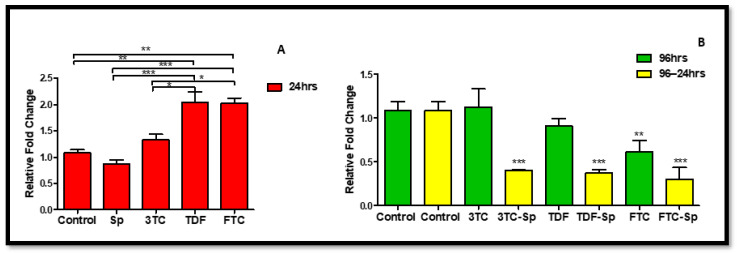
Effects of SP and HAART (3TC, TDF, and FTC) on *Akt* mRNA levels. *Akt* mRNA levels after exposure of HepG2 cells to (**A**): SP and HAART for 24 h, (**B**): HAART (96 h), and HAART (96 h) followed by SP (24 h); * p < 0.05, ** p < 0.005, *** p < 0.0001.

The previous studies showed Akt expression increased rapidly in both neurons and vascular cells following cellular stress or damage [46]. *Spirulina platensis* (SP) (24 h) and HAART (excluding 3TC) (96 h)-treated HepG2 cells expressed lower *Akt* mRNA expression B: (*p* < 0.0001), B: (*p* < 0.0001), significantly for FTC. However, as compared to the control, the HAART-SP treated cells showed a substantial reduction in *Akt* mRNA expression B: (*p* < 0.0001) (Figure 1).

### 2.2. Endothelial Nitric Oxide Synthase (eNOS)

**Figure 2:**
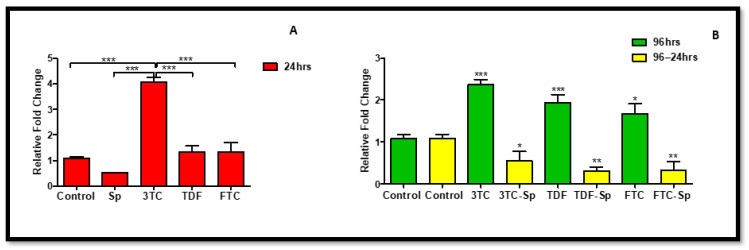
Effects of SP and HAART (3TC, TDF, and FTC) on *eNOS* mRNA levels. *eNOS* mRNA levels after exposure of HepG2 cells to (**A**): SP and HAART for 24 h, (**B**): HAART (96 h), and HAART (96 h) followed by SP (24 h); * p < 0.05, ** p < 0.005, *** p < 0.0001.

ENOS plays a role in primary hepatocytes to induce activation of the stress-responsive transcription factor, nuclear factor-erythroid 2-related factor 2 (NRF-2), under metabolic syndrome-promoting conditions [47]. The upregulation of eNOS is mostly promoted under stress conditions, such as oxidative stress and metabolic syndrome. It is noteworthy that favourable cellular conditions may not necessarily upregulate eNOS for survival or stress-related struggles. In SP- and HAART-SP-treated cells, the expression of *eNOS* mRNA was considerably reduced: A: (*p* < 0.0001), B: (*p* < 0.0001) (Figure 2). The HAART-treated cells, on the other hand, showed a significant increase in *eNOS* mRNA expression B (*p* < 0.0001) (Figure 2). 

### 2.3. The Role of Spirulina platensis in the Regulation of eNOS 

**Figure 3:**
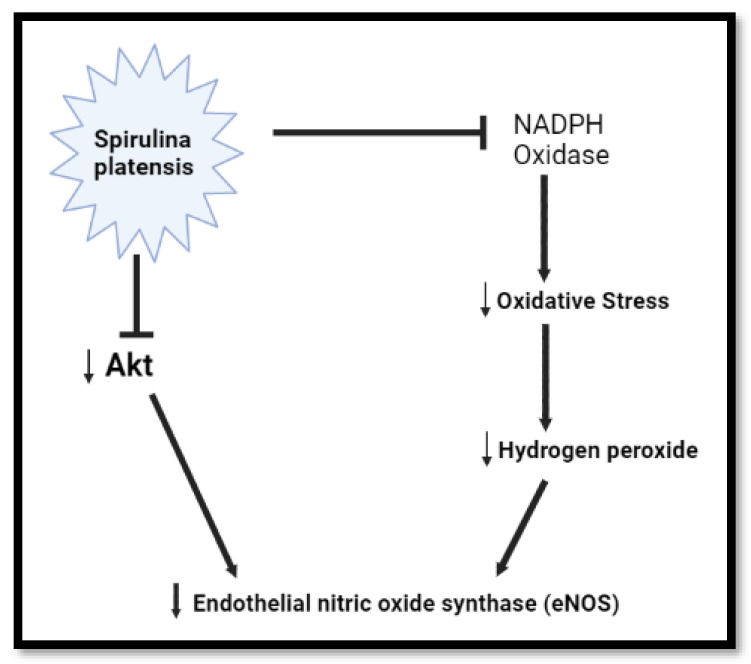
Diagrammatic representation of *Spirulina platensis* reduction of oxidative stress via inhibition of NADPH oxidase, leading up to *eNOS* mRNA downregulation. *Spirulina platensis* inhibits NADPH oxidase, reduces ROS, and blocks free radical species including H_2_O_2_, leading to the reduction of oxidative stress and reduced demand for eNOS (created with BioRender.com).

The endothelial NO synthase (eNOS) is activated by hydrogen peroxide calcium- and Akt-dependent pathways. It has been demonstrated that eNOS activation by hydrogen peroxide is accompanied by Akt activation and increased eNOS phosphorylation [48]. Moreover, under enhanced oxidative stress conditions, endothelial cells employ this mechanism to maintain NO bioactivity [48]. The NADPH oxidase (NOX) family of enzymes is the major source of reactive oxygen species (ROS) involved in both metabolic syndrome and cardiovascular pathophysiology [49,50]. Oxidative stress, which is defined as a disturbance in the balance between the ROS production and the antioxidant defence, shifted towards excess ROS, a state characterized by elevated ROS levels. ROS involves two main categories: (a) free radicals such as superoxide (O_2_^−^), hydroxyl (OH·) and nitric oxide (NO·); and (b) non-radical derivatives of O_2_, such as hydrogen peroxide (H_2_O_2_) and peroxynitrite (ONOO^−^) [51]. Oxidative stress is involved in metabolic syndrome and numerous cardiovascular diseases [51]. Normally, oxidative stress causes damage to proteins, lipids, and DNA, resulting in cellular dysfunction [52]. Akt expression is known to be upregulated rapidly in both neurons and vascular cells following cellular dysfunction [46]. Therefore, Akt does not need to be upregulated in a cellular non-threatening environment. Interestingly, the phycocyanin from *Spirulina platensis* is responsible for reducing oxidative stress and NADPH oxidase [28]. The inhibition of NADPH oxidase can also inhibit the increase of NO production through the suppression of inducible NO synthase (iNOS) [53]. However, it has been demonstrated that *Spirulina platensis* promotes the activation and expression of endothelial nitric oxide synthase (eNOS) protein under cellular dysfunction, such as atherosclerotic vascular disease [54]. Herein, *Spirulina platensis* downregulated the mRNA expression of eNOS; this might be due to the fact that *Spirulina platensis* promotes a healthy environment by reducing oxidative stress, which may result in the downregulation of genes that are mostly initiated by cellular dysfunction, such as *Akt* and *eNOS* (Figure 3).

### 2.4. Antioxidant Response

**Figure 4:**
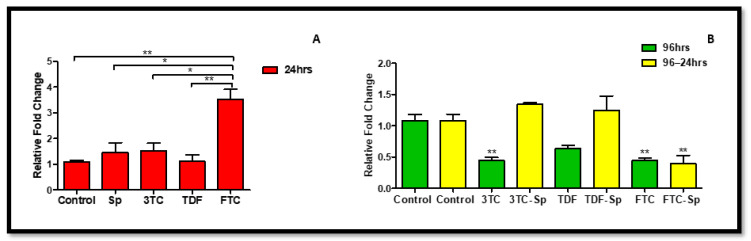
Effects of SP and HAART (3TC, TDF, and FTC) on *NRF-2* mRNA levels. *NRF-2* mRNA levels after exposure of HepG2 cells to (**A**): SP and HAART for 24 h, (**B**): HAART (96 h), and HAART (96 h) followed by SP (24 h); * p < 0.05, ** p < 0.005.

*NRF-2* mRNA levels rose in SP- and HAART-SP-treated cells (A: *p* = 0.0021 and B: *p* < 0.0001, respectively) (Figure 4). *NRF-2* mRNA expression, on the other hand, was reduced in HAART-treated cells after prolonged exposure B: (*p* < 0.0001) (Figure 4).

#### 2.4.1. Keap1 Response

**Figure 5:**
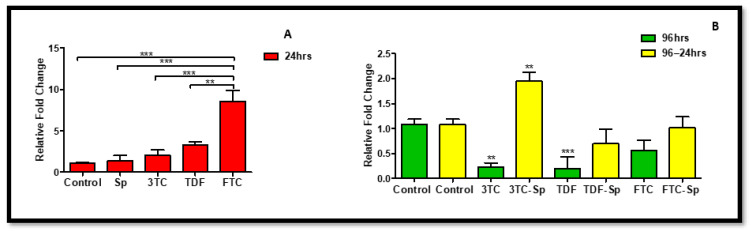
Effects of SP and HAART (3TC, TDF, and FTC) on *Keap1* mRNA levels. *Keap1* mRNA levels after exposure of HepG2 cells to (**A**): SP and HAART for 24 h, (**B**): HAART (96 h), and HAART (96 h) followed by SP (24 h); ** p < 0.005, *** p < 0.0001.

Keap1 controls the stability and cellular distribution of NRF-2. SP-and HAART-SP (excluding TDF-SP)-treated cells had higher levels of *Keap1* mRNA expression A: (*p* = 0.0002), B: (*p* < 0.0001). The HAART-treated cells, on the other hand, showed a substantial reduction in Keap1 mRNA expression after prolonged exposure B: (*p* < 0.0001) (Figure 5).

#### 2.4.2. NQO-1 Response

**Figure 6:**
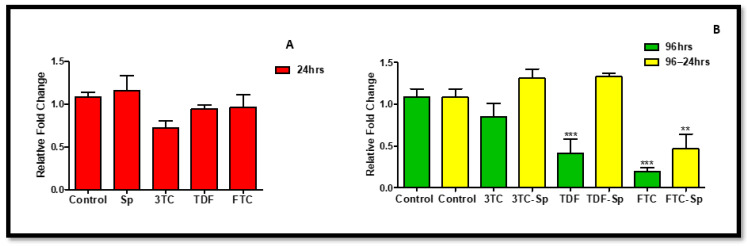
Effects of SP and HAART (3TC, TDF, and FTC) on *NQO-1* mRNA levels. *NQO-1* mRNA levels after exposure of HepG2 cells to (**A**): SP and HAART for 24 h, (**B**): HAART (96 h), and HAART (96 h) followed by SP (24 h); ** p < 0.005, *** p < 0.0001.

The oxidative stress response caused NQO-1 to be elevated. *NQO-1* mRNA expression increased in SP-and HAART-SP (excluding FTC-SP)-treated cells A: (*p* = 0.1432), B: (*p* < 0.0001) (Figure 5), but not in HAART-treated cells (Figure 6). 

### 2.5. Detoxification of Peroxides

**Figure 7:**
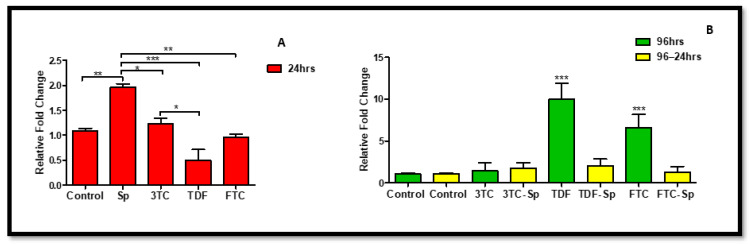
Effects of SP and HAART (3TC, TDF, and FTC) on *CAT* mRNA levels. *CAT* mRNA levels after exposure of HepG2 cells to (**A**): SP and HAART for 24 h, (**B**): HAART (96 h), and HAART (96 h) followed by SP (24 h); * p < 0.05, ** p < 0.005, *** p < 0.0001.

The redox equilibrium is maintained by catalase (CAT). *CAT* mRNA expression rose considerably in SP-and HAART-SP-treated cells, although more significantly in HAART-treated cells A: (*p* < 0.0001), B: (*p* < 0.0001) (See Figure 7). 

#### NRF-2 Protein Response

**Figure 8:**
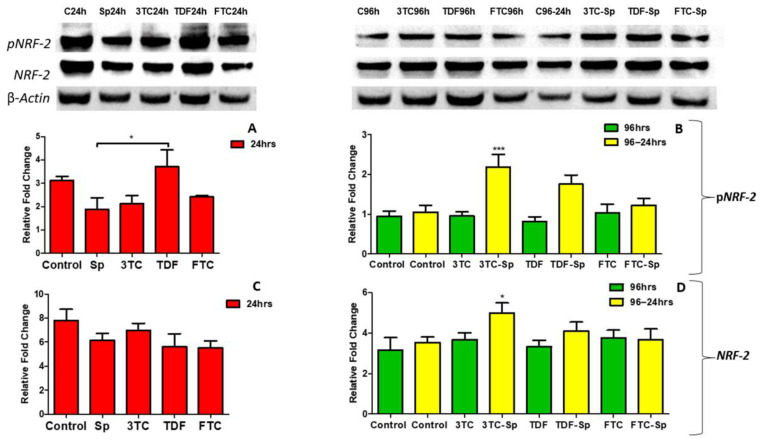
Effects of SP and HAART (3TC, TDF, and FTC) on pNRF-2 and NRF-2 protein expression. pNRF-2 protein expression after exposure of HepG2 cells to (**A**): SP and HAART for 24 h, (**B**): HAART (96 h), and HAART (96 h) followed by SP (24 h); NRF-2 protein expression after exposure of HepG2 cells to (**C**): SP and HAART for 24 h, (**D**): HAART (96 h), and HAART (96 h) followed by SP (24 h); * p < 0.05, *** p < 0.0001.

NRF-2 is a master regulator of the oxidative stress transcriptional response. The expression of the pNRF-2 protein was reduced in the SP-treated cells A: (*p* = 0.0203) (Figure 8). However, pNRF-2 protein expression was increased in the HAART- and HAART-SP-treated cells B: (*p* < 0.0001) (Figure 8). The NRF-2 protein expression was shown to be lower in SP-treated cells C: (*p* = 0.1226) after acute exposure, higher in HAART-treated cells D: (*p* = 0.0085), and considerably higher in HAART-SP-treated cells, following prolonged exposure D: (*p* = 0.0085) (Figure 8). 

## 3. Discussion

Several types of HAART drugs are commonly used in combination against HIV and these backbone drugs (3TC, TDF, and FTC) have been implicated in oxidative stress and mitochondrial toxicity [55,56,57,58]. Oxidative stress and mitochondrial toxicity are characteristic pathologies of metabolic syndrome. Metabolic syndrome effects may be ameliorated by antioxidants such as *Spirulina platensis*. *Spirulina platensis* has been reported to inhibit oxidative stress [23,26,28,29], and is highly recommended to promote mitochondrial health [30,31,32,33]. We investigated the effect of *Spirulina platensis* on the oxidative pathway after exposure of HepG2 cells to the HAART backbone (3TC, TDF, and FTC). *Spirulina platensis* exerts direct antioxidant activity and modulation of the antioxidant response element (ARE)/NRF-2 pathway in HepG2 cells [59].

In the early in vitro toxicity evaluation for pharmaceutical development, HepG2 cells are good models for analysis [60]. The liver is at significant risk of oxidative insults since the hepatocytes are rich in mitochondria and are the oxidative centre for several metabolic reactions [61]. Akt and eNOS can regulate NRF-2 activation independently and sequentially, breaking the Keap1/NRF-2 complex [62]. Akt is responsible for the activation of eNOS which takes part in the dissociation of the keap1/NRF-2 complex. The phosphorylation of NRF-2 triggers its disassociation from Keap1 ubiquitination, allowing it to translocate to the nucleus and promote the transcription of antioxidants [63]. The pNRF-2 translocates to the nucleus. In the nucleus, the pNRF-2 forms a complex with an antioxidant response element (ARE) and activates the antioxidant genes such as catalase (CAT) and NQO-1 [64].

**Figure 9:**
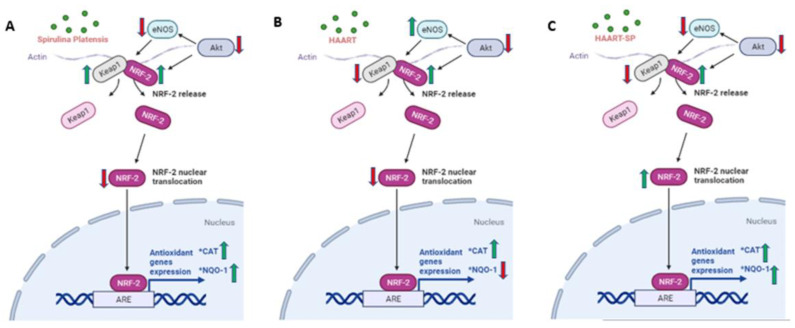
Effects of *Spirulina platensis* and HAART (3TC, TDF, and FTC) on antioxidant induction pathway in HepG2 cells; (**A**): the effect of *Spirulina platensis*; (**B**): the effect of HAART; (**C**): the effect of HAART-SP (created with BioRender.com).

*Spirulina platensis* is rich in antioxidant properties [24,27], and also contains phycocyanin, responsible for reducing oxidative stress and NADPH oxidase [28]. The antioxidant response properties of *Spirulina platensis* are evident by the significantly increased expression of *CAT* mRNA (Figure 7A) and the elevated expression of NRF-2 (Figure 4A) and NQO-1 (Figure 6A) mRNAs. In the presence of *Spirulina platensis*, the expression of *Akt* and *eNOS* (Figure 1A and Figure 2A) was reduced. The reduction of Akt may be an indication that *Spirulina platensis* does not induce cellular damage, since increased expression of Akt commonly occurs following cellular stress or damage [46]. The reduction in *eNOS* expression agrees with the reduction in *Akt* expression, as Akt is responsible for its activation. These results suggest that *Spirulina platensis* does not induce cellular stress that might require eNOS to activate the stress-responsive transcription factor NRF-2 in the hepatocytes [47]. Hence, the increased expression of *Keap1* (Figure 5A) may have prevented NRF-2 phosphorylation, as seen by the reduction in the pNRF-2 expression (Figure 8A) in the presence of only *Spirulina platensis.* Herein, the Keap1/NRF-2 complex was not dissociated by Akt or eNOS (Figure 9A). However, Strasky et al. (2013) demonstrated that *Spirulina platensis* promotes the activation and expression of eNOS protein under cellular dysfunction such as atherosclerotic vascular disease [54]. Moreover, Strasky et al. (2013) investigated protein expression, while in this present study we investigated mRNA expression. 

Interestingly, *Spirulina platensis* also contains vitamin D3, vitamin C, and magnesium, whose components have been suspected to stimulate NO production [65]. Moreover, this makes sense, since *Spirulina platensis* also contains total essential amino acids, with an abundance of arginine [66,67,68]. Nitric Oxide can modify the cysteine residues on Keap1 to augment NRF-2 activity [62]. Total *NRF-2* was upregulated at the gene level (Figure 4A); hence, detoxification antioxidant genes such as CAT and NQO-1 were upregulated. Enhanced NRF-2/ARE activation results in decreased ROS production and an enhanced concentration of antioxidant glutathione (GSH) [62]. The decreased NRF-2 protein expression (Figure 8C) makes sense, since there was an increased *Keap1* mRNA expression (Figure 5A). NRF-2 transcriptional activity is low when Keap1 is upregulated [62]. *Spirulina platensis* managed to significantly increase *CAT* mRNA expression (Figure 7A). CAT plays a major role in the detoxification of ROS products [69,70]. Previous studies have shown that NRF-2 activation by *Spirulina platensis* results in the production and increased expression of antioxidant enzymes such as catalase (CAT) [71]. *Spirulina platensis* also increased the expression of the antioxidant gene, *NQO-1* (Figure 6A).

In the presence of HAART, Akt mRNA (Figure 1B) expression varied, elevated in the presence of 3TC, and reduced in the presence of TDF and FTC. TDF is associated with the inhibition of Akt [72,73]. The presence of FTC and TDF is linked to an increased eNOS expression and a decreased Akt expression in adult rats treated with FTC-TDF-EFV [74]. In addition, this agrees with the significantly increased *eNOS* expression (Figure 2B) observed in this present study. ENOS increases NO, and breaks the Keap1/NRF-2 complex, phosphorylating NRF-2 [62]. This is evident by the elevated NRF-2 protein expression (Figure 8D) and reduced *Keap1* mRNA expression (Figure 5B). However, the *NRF-2* mRNA (Figure 4B) non-significantly decreased and the pNRF-2 expression varied, with only FTC inducing an increase in the pNRF-2 expression (Figure 8B). HAART exhausted the NRF-2 response after chronic exposure in HepG2 cells, which may explain the downregulation of pNRF-2 (Figure 8B), but the upregulation of NRF-2 at the protein level (Figure 8D) and not at the gene level (Figure 4B). Post-HAART, *CAT* was upregulated (Figure 7B), but *NQO-1* was downregulated (Figure 6B), which means HAART induced a limited antioxidant response in HepG2 cells. The upregulation of CAT makes sense, since HAART has been linked to the restoration and expression of CAT in HIV-seropositive individuals on HAART [75] (Figure 9B).

HAART-SP exposure reduced Akt and eNOS expression (Figure 1B and Figure 2B); this is an indication that there is reduced or no cellular stress [47], which may be due to post-exposure to *Spirulina platensis*. NRF-2 is a master regulator of the transcriptional response to oxidative stress [76]. Post-treatment with *Spirulina platensis* after HAART upregulated total NRF-2 and pNRF-2 (Figure 4B, Figure 8D and Figure 8B, respectively), which is the key to the activation of detoxification antioxidant genes. Hence, *CAT* and *NQO-1* were also upregulated (Figure 6B and Figure 7B). The increase in NRF-2 expression is a sign of antioxidant response and a good indication that the balance is restored, or in the process of restoration, reducing the oxidative stress. NRF-2, apart from being an antioxidant, plays a huge role in metabolic homeostasis, eliminating lipid accumulation and oxidative stress [77,78]. Eliminating oxidative stress may be the key to inhibiting hypertension and type 2 diabetes, as oxidative stress is suspected to be a major contributor to the development of metabolic syndromes [79]. Some studies have demonstrated that NRF-2 activation prevents ROS-induced damage in the pancreatic β cells [80,81], and this protective characteristic can inhibit diabetes mellitus. NRF-2 controls downstream genes, including NQO-1 antioxidant. NQO-1 reduction or knockout can result in an increase in insulin resistance in mice [82]. The presence of FTC-SP upregulated NRF-2 and pNRF-2 at the protein level, however, it downregulated *NRF-2* at the gene level. This may be due to high FTC toxicity compared to the other ARVs; more exposure time to *Spirulina platensis* may be required to ameliorate FTC toxicity or to trigger an NRF-2 response. It is noteworthy that fluoride toxicity may add to FTC adverse drug reactions [83]. The FTC persisted with the same pattern as in the cells, where *Spirulina platensis* was not introduced. In both events, the FTC reduced *NRF-2* at the gene level and downregulated *NQO-1*. NQO-1 is a phase-II detoxifying enzyme that uses the pyridine nucleotides NADPH or NADH as an electron donor to reduce quinones to hydroquinones. Additionally, NQO-1 reduces ROS production via direct scavenging superoxide [62] (Figure 9C). The data above implies that the *Spirulina platensis* and HAART synergy may be via an NRF-2 pathway response.

## 4. Materials and Methods

### 4.1. Materials

*Spirulina platensis*, extracted from *Spirulina platensis* (Shewal) capsules, was obtained from HeriCure Healthcare Ltd. (Pune, India); A 10 mg/mL aqueous stock solution of the extract was prepared from the capsule content (the *Spirulina platensis* from the capsules was dissolved in distilled water (dH_2_O)), and the solution was filtered (0.45 μm) and used to prepare the concentrations of *Spirulina platensis* extract required for the study). The extract was then incubated at −80 °C for 24 h and lyophilized for 48 h using the SP Vir Tis Scientific Freeze Dryer (Warminster, PA, USA) (−46 °C, 79 mT,). The final weight of the extracts was obtained, and the extracts were stored in the dark at 4 °C until further use. For heat-sensitive cyanobacteria, such as *spirulina*, freeze-drying is one of the best treatment options since it causes the least amount of changes to their nutritive, sensory, and physicochemical characteristics, leaving the lyophilized products similar to the fresh biomass [40]. The antiretroviral drug compounds were purchased from Pharmed Pharmaceuticals and extracted using dichloromethane, which was then removed using a standard laboratory rotary evaporator. The identity of the extracted compounds was confirmed using NMR analysis and showed a purity of >98%. The resultant antiretroviral drugs were obtained from the NIH AIDS Reagents Program. The HepG2 cell line was acquired from Highveld Biologicals (Johannesburg, South Africa). The cell culture reagents and supplements were purchased from Lonza BioWhittaker (Basel, Switzerland). Unless otherwise stated, all the other used reagents were purchased from Merck (Darmstadt, Germany).

### 4.2. Cell Culture

The HepG2 cells were cultured in a monolayer (10^6^ cells per 25 cm^3^ culture flask) with complete culture media [CCM: Eagle’s Essential Minimal Media (EMEM) supplemented with 10% foetal calf serum, 1% penstrepfungizone and 1% L-glutamine] at 37 °C in a humidified incubator. The cells were allowed to reach 80% confluence in 25 cm^3^ flasks before treatment with only antiretrovirals (ARVs), using the plasma peak values from the literature that represent the physiological concentrations of ARVs in humans (3TC: 6.6 µM (1.51 µg/mL), TDF: 0.3 µg/mL, and FTC: 1.8 µg/mL) [37,38,39] in CCM for 96 h [84]. For the 96 h of treatment, fresh cell culture medium containing ARVs treatment was replenished every 48 h. Thereafter, the ARVs were removed, and the cells were gently rinsed with 0.1 mol/L phosphate buffer saline (PBS) and treated with only 1.5 µg/mL *Spirulina platensis* (SP) in CCM for 24 h. An untreated control, containing only CCM, was also prepared. A treatment for a 24-h time period was also conducted, containing only ARVs [85] and SP separately.

### 4.3. Protein Expression

The protein expressions of nuclear factor-erythroid 2-related factor 2 (NRF-2) and phosphorylated NRF-2 (p NRF-2) were determined by Western blotting. Standardised protein samples were boiled in Laemmeli buffer [dH_2_O, 0.5 mol/L Tris-HCl (pH 6.8), glycerol, 10% sodium dodecyl sulphide (SDS), and b-mercaptoethanol, 1% bromophenol blue] for 5 min. The proteins (25 μL) were separated by electrophoresis on SDS-polyacrylamide electrophoresis gels (4% stacking gel; 10% resolving gel) and electrotransferred to nitrocellulose membranes. The membranes were blocked with 5% BSA in Tween 20-Tris buffer saline (TTBS 150 mmol/L NaCl, 3 mmol/L KCl, 25 mmol/L Tris, 0.05% Tween 20, and dH_2_O, pH 7.5) for 1 h and incubated with a primary antibody [pNRF-2 (ab76026); NRF-2 (ab31163)] in 5% BSA in TTBS (1:1000 dilution) overnight at 4 °C. Following the overnight incubation, the membranes were equilibrated to room temperature (RT) and washed with TTBS (5 times, 10 min). The membranes were subsequently probed with a horseradish peroxidase (HRP)-conjugated secondary antibody [Rabbit (7074S)] in 5% BSA in TTBS (1:5000) for 1 h at RT. Thereafter, the membranes were washed with TTBS (5 times, 10 min) and immunoreactivity was detected (Clarity Western ECL Substrate) with the Bio-Rad ChemiDoc gel documentation system. After detection, the membranes were quenched with 5% H_2_O_2_ for 30 min, then rinsed once in TTBS and incubated in a blocking solution (5% BSA for 1 h at RT), rinsed thrice in TTBS, and probed with HRP-conjugated anti-β-actin (housekeeping protein). The protein expression was analysed by the Image Lab Software version 5.0 (Bio-Rad) and the results were expressed as the relative band density (RBD). The expression of proteins of interest was normalized against β-Actin.

### 4.4. RNA Analysis

Total ribonucleic acid (RNA) was isolated according to the method described by Chuturgoon, Phulukdaree and Moodley [86]. Isolated RNA was quantified (Nanodrop 2000, Thermo Scientific, Waltham, MA, USA) and standardized to 1000 ng/μL. Complementary deoxyribonucleic acid (cDNA) was synthesized from the standardized RNA using the iScript cDNA synthesis kit (Bio-Rad). The thermocycler conditions for the cDNA synthesis were 25 °C for 5 min, 42 °C for 30 min, 85 °C for 5 min, and a final hold at 4 °C [85]. The gene expression was analysed using the SsoAdvanced Universal SYBR Green Supermix kit (Bio-Rad). The messenger RNA (mRNA) expressions of *NRF-2*, *Kelch-like ECH-associated protein 1 (Keap1)*, *catalase (CAT)*, *NADH quinone oxidoreductase 1 (NQO-1)*, *Endothelial nitric oxide synthase* (*eNOS)*, and *protein kinase B (Akt)* were investigated using specific forward and reverse primers (Table 1). The reaction volumes, which consisted of the following, were prepared: SYBR green (5 μL), forward primer (1 μL), reverse primer (1 μL), nuclease-free water (2 μL), and the cDNA template (1 μL). All the reactions were carried out in triplicate. The samples were amplified using a CFX96 TouchReal-Time PCR Detection System (Bio-Rad). The initial denaturation occurred at 95 °C (4 min). Thereafter, 37 cycles of denaturation (15 s, 95 °C), annealing (40 s; temperatures—Table 1), and extension (30 s, 72 °C) occurred. The method described by Livak and Schmittgen [87] was employed to determine the changes in relative mRNA expression, where 2^−ΔΔCt^ represents the fold change relative to the untreated control. The expression of the gene of interest was normalized against the housekeeping gene, Glyceraldehyde 3-phosphate dehydrogenase (GAPDH), which was amplified simultaneously under the same conditions.

### 4.5. Statistical Analysis

All the experiments were conducted independently in triplicate. GraphPad Prism version 5.0 (GraphPad Software Inc., San Diego, CA, USA) was used to perform all the statistical analyses. The one-way analysis of variance (ANOVA), followed by a Bonferroni test for multiple group comparison (data are presented as 95% CI), was used to determine statistical significance. All the results were represented as the mean ± standard deviation unless otherwise stated. A value of *p* < 0.05 was considered statistically significant.

## 5. Conclusions

*Spirulina platensis,* as an antioxidant and anti-inflammatory agent, possesses various corrective properties against HAART adverse drug reactions. The corrective properties of *Spirulina platensis* shown in this study highlight its potential to mitigate HAART adverse drug reactions. This study investigated the antioxidant properties of the potent antioxidant, *Spirulina platensis.* In addition, the study also explored how *Spirulina platensis* supplementation may benefit HAART-dependent individuals. The results revealed that *Spirulina platensis* ameliorated HAART toxicity via the induction of an antioxidant response. In addition, the study showed a significant response to the positive synergistic HAART-SP effect theory. Individuals on HAART may benefit from *Spirulina platensis* supplementation. However, due to the fluoride content of FTC, a well-known mitochondrial toxicity-inducing compound, we recommend more studies on FTC toxicity. Furthermore, we suggest the need for further studies on the *Spirulina platensis* and HAART synergy. 

## Figures and Tables

**Table 1:** The annealing temperatures and primer sequences for the genes of interest.

Gene	Annealing Temperature	Primer	Sequence	Length	Accession Number	Product Length
*Nrf-2*	60 °C	Forward Reverse	5′-CACATCCAGTCAGAAACCAGTGG-3′	2446	NM_006164.5	772 bp
			5′-GGAATGTCTGCGCCAAAAGCTG-3′			
*Keap1*	59.3 °C	Forward Reverse	5′-CTGGAGGATCATACCAAGCAGG-3′	2524	NM_012289.4	1599 bp
			5′-GGATACCCTCAATGGACACCAC-3′			
*CAT*	53 °C	Forward Reverse	5′-GTGCGGAGATTCAACACTGCCA-3′	2291	NM_001752.4	1043 bp
			5′-CGGCAATGTTCTCACACAGACG-3′			
*NQO-1*	59.7 °C	Forward Reverse	5′-GAAGAGCACTGATCGTACTGGC-3′	2407	NM_001025434.2	1907 bp
			5′-GGATACTGAAAGTTCGCAGGG-3′			
*eNOS*	59.7 °C	Forward Reverse	5′-GTGGCTGTCTGCATGGACCT-3′	2058	NM_001160109.2	1788 bp
			5′-CCACGATGGTGATTTGGCT-3′			
*Akt*	59.7 °C	Forward Reverse	5′-TGGACTACCTGCACTCGGAGAA-3′	2922	NM_001382431.1	1468 bp
			5′-GTGCCGCAAAAGGTCTTCATGG-3′			
*GAPDH*	Same as the gene of interest	Forward Reverse	5′-TCCACCACCCTGTTGCTGTA-3′	1285	NM_002046.7	231 bp
			5′-ACCACAGTCCATGCCATCAC-3′			

## Data Availability

Not applicable.

## Abbreviations

acquired immunodeficiency syndrome	AIDS
antioxidant response element	ARE
antiretrovirals	ARVs
Catalase	CAT
Complementary deoxyribonucleic acid	cDNA
Complete culture media	CCM
distilled water	dH_2_O
Eagle’s Essential Minimal Media	EMEM
Emtricitabine/2′,3′-dideoxy-5fluoro-3′-thiacytidine	FTC
Endothelial nitric oxide synthase	eNOS
Glyceraldehyde 3-phosphate dehydrogenase	GAPDH
highly active antiretroviral therapy	HAART
human immunodeficiency virus	HIV
Human hepatoma/Human liver cells	HepG2
horseradish peroxidase	HRP
hydroxyl	OH·
hydrogen peroxide	H_2_O_2_
inducible NO synthase	iNOS
tenofovir disoproxil fumarate	TDF
Kelch-like ECH-associated protein 1	Keap1
Lamivudine/(-)-L-2′,3′-dideoxy-3′-thiacytidine	3TC
Messenger RNA	mRNA
nuclear factor erythroid 2-related factor 2	NRF-2
nucleoside/nucleotide reverse transcriptase inhibitors	NRTIs
NADH quinone oxidoreductase 1	NQO-1
NADPH oxidase	NOX
nitric oxide	NO·
Nuclear Magnetic Resonance	NMR
One-way analysis of variance	ANOVA
Phosphate buffered saline	PBS
phosphorylated NRF-2	pNRF-2
potential exposure prophylaxis	PEP
Pre-exposure prophylaxis	PrEP
protein kinase B	Akt
Quantitative polymerase chain reaction	qPCR
Reactive oxygen species	ROS
relative band density	RBD
ribonucleic acid	RNA
room temperature	RT
Spirulina platensis	SP
Sodium dodecyl sulphide	SDS
tenofovir disoproxil fumarate	TDF
World Health Organization	WHO

## References

[1] (2021). WHO, HIV/AIDS. https://www.who.int/news-room/fact-sheets/detail/hiv-aids.

[2] Release S. Mid-Year Population Estimates. 2021, Statistics South Africa. http://www.statssa.gov.za/publications/P0302/P03022021.pdf.

[3] UNAIDS (2021). Joint United Nations Programme on HIV/AIDS (UNAIDS). Global Report: UNAIDS Report on the Global AIDS Epidemic 2021.

[4] UNAIDS-Global HIV & AIDS Statistics—2020 Fact Sheet. https://www.unaids.org/en/resources/fact-sheet.

[5] Africa U.-S. (2020). HIV and AIDS Estimates. https://www.unaids.org/en/regionscountries/countries/southafrica.

[6] National Institute on Drug Abuse (NIDA) What is HAART? 2012. https://www.https://www.drugabuse.gov/drugabuse.gov/publications/research-reports/hivaids/what-haart.

[7] Brouwer E.S., Napravnik S., Eron J.J., Stalzer B., Floris-Moore M., Simpson R.J., Stürmer T. (2014). Effects of Combination Antiretroviral Therapies on the Risk of Myocardial Infarction Among HIV Patients. Epidemiology.

[8] Mohan J., Ghazi T., Chuturgoon A.A. (2021). A Critical Review of the Biochemical Mechanisms and Epigenetic Modifications in HIV- and Antiretroviral-Induced Metabolic Syndrome. Int. J. Mol. Sci..

[9] Rakhmanina N.Y., la Porte C.J. (2012). Therapeutic drug monitoring of antiretroviral drugs in the management of human immunodeficiency virus infection. Ther. Drug Monit. Newer Drugs Biomark..

[10] EACS (2011). European AIDS Clinical Society Guidelines. Amsterdam, The Netherlands, Version 6. https://www.eacsociety.org/media/2011_eacsguidelines-v6.0-english_oct.pdf.

[11] GGilks C.F., Crowley S., Ekpini R., Gove S., Perriens J., Souteyrand Y., Sutherland D., Vitoria M., Guerma T., De Cock K. (2006). The WHO public-health approach to antiretroviral treatment against HIV in resource-limited settings. Lancet.

[12] Kahana S.Y., Rohan J., Allison S., Frazier T.W., Drotar D. (2012). A Meta-Analysis of Adherence to Antiretroviral Therapy and Virologic Responses in HIV-Infected Children, Adolescents, and Young Adults. AIDS Behav..

[13] Sliwa K., Hilfiker-Kleiner D., Petrie M.C., Mebazaa A., Pieske B., Buchmann E., Regitz-Zagrosek V., Schaufelberger M., Tavazzi L., van Veldhuisen D.J. (2010). Current state of knowledge on aetiology, diagnosis, management, and therapy of peripartum cardiomyopathy: A position statement from the Heart Failure Association of the European Society of Cardiology Working Group on peripartum cardiomyopathy. Eur. J. Hear. Fail..

[14] Mugwanya K.K., John-Stewart G., Baeten J. (2017). Safety of oral tenofovir disoproxil fumarate-based HIV pre-exposure prophylaxis use in lactating HIV-uninfected women. Expert Opin. Drug Saf..

[15] Spinner C., Boesecke C., Zink A., Jessen H., Stellbrink H.-J., Rockstroh J.K., Esser S. (2015). HIV pre-exposure prophylaxis (PrEP): A review of current knowledge of oral systemic HIV PrEP in humans. Infection.

[16] Fonner V.A., Dalglish S.L., Kennedy C.E., Baggaley R., O’reilly K.R., Koechlin F.M., Rodolph M., Hodges-Mameletzis I., Grant R.M. (2016). Effectiveness and safety of oral HIV preexposure prophylaxis for all populations. AIDS.

[17] Maserati R., De Silvestri A., Uglietti A., Colao G., Di Biagio A., Bruzzone B., Di Pietro M., Re M.C., Tinelli C., Zazzi M. (2010). Emerging mutations at virological failure of HAART combinations containing tenofovir and lamivudine or emtricitabine. AIDS.

[18] Borroto-Esoda K., Vela J.E., Myrick F., Ray A.S., Miller M.D. (2005). In Vitro Evaluation of the Anti-HIV Activity and Metabolic Interactions of Tenofovir and Emtricitabine. Antivir. Ther..

[19] Mujugira A., Baeten J.M., Hodges-Mameletzis I., Haberer J.E. (2020). Lamivudine/Tenofovir Disoproxil Fumarate is an Appropriate PrEP Regimen. Drugs.

[20] Cheney L., Barbaro J., Berman J. (2021). Antiretroviral Drugs Impact Autophagy with Toxic Outcomes. Cells.

[21] Milián L., Peris J.E., Gandía P., Andújar I., Pallardó L., Górriz J.L., Blas-García A. (2017). Tenofovir-induced toxicity in renal proximal tubular epithelial cells: Involvement of mitochondria. AIDS.

[22] Nelson M., Schiavone M. (2004). Emtricitabine (FTC) for the treatment of HIV infection. Int. J. Clin. Pract..

[23] Zheng J., Inoguchi T., Sasaki S., Maeda Y., McCarty M.F., Fujii M., Ikeda N., Kobayashi K., Sonoda N., Takayanagi R. (2013). Phycocyanin and phycocyanobilin from Spirulina platensis protect against diabetic nephropathy by inhibiting oxidative stress, American Journal of Physiology-Regulatory. Integr. Comp. Physiol..

[24] Hu Z.H., Liu Z.L. (2001). Determination and purification of beta-carotene in Spirulina maximum. Chin. J. Chromatogr..

[25] Miranda M., Cintra R., Barros S., Mancini-Filho J. (1998). Antioxidant activity of the microalga Spirulina maxima. Braz. J. Med. Biol. Res..

[26] Bashandy S.A.E., El Awdan S.A., Ebaid H., Alhazza I.M. (2016). Antioxidant Potential of *Spirulina platensis* Mitigates Oxidative Stress and Reprotoxicity Induced by Sodium Arsenite in Male Rats. Oxidative Med. Cell. Longev..

[27] Estrada J.P., Bescós P.B., del Fresno A.V. (2001). Antioxidant activity of different fractions of Spirulina platensis protean extract. Farmaco.

[28] Riss J., Décordé K., Sutra T., Delage M., Baccou J.-C., Jouy N., Brune J.-P., Oréal H., Cristol J.-P., Rouanet J.-M. (2007). Phycobiliprotein C-Phycocyanin from *Spirulina platensis* Is Powerfully Responsible for Reducing Oxidative Stress and NADPH Oxidase Expression Induced by an Atherogenic Diet in Hamsters. J. Agric. Food Chem..

[29] Abdelkhalek N.K.M., Ghazy E.W., Abdel-Daim M.M. (2014). Pharmacodynamic interaction of Spirulina platensis and deltamethrin in freshwater fish Nile tilapia, Oreochromis niloticus: Impact on lipid peroxidation and oxidative stress. Environ. Sci. Pollut. Res..

[30] Nawrocka D., Kornicka K., Śmieszek A., Marycz K. (2017). Spirulina platensis Improves Mitochondrial Function Impaired by Elevated Oxidative Stress in Adipose-Derived Mesenchymal Stromal Cells (ASCs) and Intestinal Epithelial Cells (IECs), and Enhances Insulin Sensitivity in Equine Metabolic Syndrome (EMS) Horses. Mar. Drugs.

[31] Jadaun P., Yadav D., Bisen P.S. (2017). Spirulina platensis prevents high glucose-induced oxidative stress mitochondrial damage mediated apoptosis in cardiomyoblasts. Cytotechnology.

[32] Sun J.Y., Hou Y.J., Fu X.Y., Fu X.T., Ma J.K., Yang M.F., Sun B.L., Fan C.D., Oh J. (2018). Selenium-Containing Protein From Selenium-Enriched Spirulina platensis Attenuates Cisplatin-Induced Apoptosis in MC3T3-E1 Mouse Preosteoblast by Inhibiting Mitochondrial Dysfunction and ROS-Mediated Oxidative Damage. Front. Physiol..

[33] Oriquat G.A., Ali M.A., Mahmoud S.A., Eid R.M., Hassan R., Kamel M.A. (2019). Improving hepatic mitochondrial biogenesis as a postulated mechanism for the antidiabetic effect of Spirulina platensis in comparison with metformin. Appl. Physiol. Nutr. Metab..

[34] Ali S.K., Saleh A.M. (2012). Spirulina-an overview. Int. J. Pharm. Pharm. Sci..

[35] Ayehunie S., Belay A., Baba T.W., Ruprecht R.M. (1998). Inhibition of HIV-1 replication by an aqueous extract of Spirulina platensis (*Arthrospira platensis*). J. Acquir. Immune Defic. Syndr. Hum. Retrovirology Off. Publ. Int. Retrovirology Assoc..

[36] Asghari A., Fazilati M., Latifi A.M., Salavati H., Choopani A. (2016). A review on antioxidant properties of Spirulina. J. Appl. Biotechnol. Rep..

[37] Uma M.I., Sophia A., Uliyar V.M. (1999). Glycemic and lipemic responses of selected spirulina-supplemented rice-based recipes in normal subjects. Age.

[38] Serban M.-C., Sahebkar A., Dragan S., Stoichescu-Hogea G., Ursoniu S., Andrica F., Banach M. (2015). A systematic review and meta-analysis of the impact of Spirulina supplementation on plasma lipid concentrations. Clin. Nutr..

[39] Finamore A., Palmery M., Bensehaila S., Peluso I. (2017). Antioxidant, immunomodulating, and microbial-modulating activities of the sustainable and ecofriendly spirulina. Oxidative Med. Cell. Longev..

[40] Stramarkou M., Papadaki S., Kyriakopoulou K., Tzovenis I., Chronis M., Krokida M. (2021). Comparative Analysis of Different Drying Techniques Based on the Qualitative Characteristics of *Spirulina platensis* Biomass. J. Aquat. Food Prod. Technol..

[41] Kannan M., Pushparaj A., Dheeba B., Nageshwari K., Kannan K. (2014). Phytochemical screening and antioxidant activity of marine algae Gracilaria corticata and Spirulina platensis. J. Chem. Pharm. Res..

[42] Jaime L., Mendiola J.A., Herrero M., Soler-Rivas C., Santoyo S., Señorans F.J., Cifuentes A., Ibáñez E. (2005). Separation and characterization of antioxidants from Spirulina platensis microalga combining pressurized liquid extraction, TLC, and HPLC-DAD. J. Sep. Sci..

[43] El-Baky A., Hanaa H., el Baz F., El-Baroty G.S. (2009). Enhancement of antioxidant production in Spirulina platensis under oxidative stress. Acta Physiol. Plant..

[44] Prabakaran G., Sampathkumar P., Kavisri M., Moovendhan M. (2020). Extraction and characterization of phycocyanin from Spirulina platensis and evaluation of its anticancer, antidiabetic and antiinflammatory effect. Int. J. Biol. Macromol..

[45] Herrero M., Vicente M.J., Cifuentes A., Ibáñez E. (2007). Characterization by high-performance liquid chromatography/electrospray ionization quadrupole time-of-flight mass spectrometry of the lipid fraction of Spirulina platensis pressurized ethanol extract, Rapid Communications in Mass Spectrometry. Int. J. Devoted Rapid Dissem. Minute Res. Mass Spectrom..

[46] Adefolaju G.A., Scholtz K.E., Hosie M.J. (2017). Expresión de VEGF165b Antiangiogénico en Células MCF-7 y MCF-10A de Mama Humana Expuesto a Inhibidores de la Transcriptasa Inversa y la Proteasa. Int. J. Morphol..

[47] Nagiah S., Phulukdaree A., Chuturgoon A. (2015). Mitochondrial and Oxidative Stress Response in HepG2 Cells Following Acute and Prolonged Exposure to Antiretroviral Drugs. J. Cell. Biochem..

[48] Chuturgoon A., Phulukdaree A., Moodley D. (2014). Fumonisin B1 induces global DNA hypomethylation in HepG2 cells—An alternative mechanism of action. Toxicology.

[49] KLivak J., Schmittgen T.D. (2001). Analysis of relative gene expression data using real-time quantitative PCR and the 2^−ΔΔCT^ method. Methods.

[50] Chong Z., Li F., Maiese K. (2005). Activating Akt and the brain’s resources to drive cellular survival and prevent inflammatory injury. Histol. Histopathol..

[51] Sheldon R., Meers G.M., Morris E.M., Linden M., Cunningham R.P., Ibdah J.A., Thyfault J.P., Laughlin M.H., Rector R.S. (2019). eNOS deletion impairs mitochondrial quality control and exacerbates Western diet-induced NASH. Am. J. Physiol. Metab..

[52] Thomas S.R., Chen K., Keaney J.F. (2002). Hydrogen Peroxide Activates Endothelial Nitric-oxide Synthase through Coordinated Phosphorylation and Dephosphorylation via a Phosphoinositide 3-Kinase-dependent Signaling Pathway. J. Biol. Chem..

[53] Fortuño A., José G.S., Moreno M.U., Beloqui O., Díez J., Zalba G. (2006). Phagocytic NADPH oxidase overactivity underlies oxidative stress in metabolic syndrome. Diabetes.

[54] Elnakish M.T., Hassanain H.H., Janssen P.M., Angelos M.G., Khan M. (2013). Emerging role of oxidative stress in metabolic syndrome and cardiovascular diseases: Important role of Rac/NADPH oxidase. J. Pathol..

[55] Paravicini T.M., Touyz R.M. (2008). NADPH oxidases, reactive oxygen species, and hypertension: Clinical implications and therapeutic possibilities. Diabetes Care.

[56] Sun M., Huang X., Yan Y., Chen J., Wang Z., Xie M., Li J. (2012). Rac1 Is a Possible Link Between Obesity and Oxidative Stress in Chinese Overweight Adolescents. Obesity.

[57] Bai S.-K., Lee S.-J., Na H.-J., Ha K.-S., Han J.-A., Lee H., Kwon Y.-G., Chung C.-K., Kim Y.-M. (2005). β-Carotene inhibits inflammatory gene expression in lipopolysaccharide-stimulated macrophages by suppressing redox-based NF-κB activation. Exp. Mol. Med..

[58] Strasky Z., Zemankova L., Nemeckova I., Rathouska J., Wong R.J., Muchova L., Subhanova I., Vanikova J., Vanova K., Vitek L. (2013). Spirulina platensis and phycocyanobilin activate atheroprotective heme oxygenase-1: A possible implication for atherogenesis. Food Funct..

[59] Glover M., Hebert V.Y., Nichols K., Xue S.Y., Thibeaux T.M., Zavecz J.A., Dugas T.R. (2014). Overexpression of mitochondrial antioxidant manganese superoxide dismutase (MnSOD) provides protection against AZT- or 3TC-induced endothelial dysfunction. Antivir. Res..

[60] Brown L., Jin J., Ferrell D., Sadic E., Obregon D., Smith A.J., Tan J., Giunta B. (2014). Efavirenz Promotes β-Secretase Expression and Increased Aβ1-40,42 via Oxidative Stress and Reduced Microglial Phagocytosis: Implications for HIV Associated Neurocognitive Disorders (HAND). PLoS ONE.

[61] Canale D., De Bragança A.C., Gonçalves J.G., Shimizu M.H.M., Sanches T.R., Andrade L., Volpini R.A., Seguro A.C. (2014). Vitamin D Deficiency Aggravates Nephrotoxicity, Hypertension and Dyslipidemia Caused by Tenofovir: Role of Oxidative Stress and Renin-Angiotensin System. PLoS ONE.

[62] Venhoff N., Setzer B., Melkaoui K., Walker U. (2007). Mitochondrial Toxicity of Tenofovir, Emtricitabine and Abacavir Alone and in Combination with Additional Nucleoside Reverse Transcriptase Inhibitors. Antivir. Ther..

[63] Vigliante I., Mannino G., Maffei M.E. (2019). OxiCyan®, a phytocomplex of bilberry (*Vaccinium myrtillus*) and spirulina (*Spirulina platensis*), exerts both direct antioxidant activity and modulation of ARE/Nrf2 pathway in HepG2 cells. J. Funct. Foods.

[64] Westerink W.M., Schoonen W.G. (2007). Cytochrome P450 enzyme levels in HepG2 cells and cryopreserved primary human hepatocytes and their induction in HepG2 cells. Toxicol. Vitr..

[65] Johannsen D.L., Ravussin E. (2009). The role of mitochondria in health and disease. Curr. Opin. Pharmacol..

[66] McSweeney S.R., Warabi E., Siow R.C. (2016). Nrf2 as an endothelial mechanosensitive transcription factor: Going with the flow. Hypertension.

[67] Huang H.-C., Nguyen T., Pickett C.B. (2002). Phosphorylation of Nrf2 at Ser-40 by Protein Kinase C Regulates Antioxidant Response Element-mediated Transcription. J. Biol. Chem..

[68] Nguyen T., Nioi P., Pickett C.B. (2009). The Nrf2-Antioxidant Response Element Signaling Pathway and Its Activation by Oxidative Stress. J. Biol. Chem..

[69] Houston M.C. (2013). The role of nutrition and nutraceutical supplements in the prevention and treatment of hypertension. Clin. Pr..

[70] Al-Batshan H.A., Al-Mufarrej S.I., Al-Homaidan A.A., Qureshi M. (2001). Enhancement of chicken macrophage phagocytic function and nitrite production by dietary Spirulina platensis. Immunopharmacol. Immunotoxicol..

[71] Sun Y., Chang R., Li Q., Li B. (2015). Isolation and characterization of an antibacterial peptide from protein hydrolysates of Spirulina platensis. Eur. Food Res. Technol..

[72] El-Sayed A.E.-K., Mostafa E.-S. (2018). Outdoor cultivation of Spirulina platensis for mass production. Not. Sci. Biol..

[73] Krinsky N.I. (1992). Mechanism of action of biological antioxidants. Proc. Soc. Exp. Biol. Med..

[74] Wang X., Wu Q., Wan D., Liu Q., Chen D., Liu Z., Martínez-Larrañaga M.R., Martínez M.A., Anadón A., Yuan Z. (2016). Fumonisins: Oxidative stress-mediated toxicity and metabolism in vivo and in vitro. Arch. Toxicol..

[75] Sadek K.M., Lebda M.A., Nasr S.M., Shoukry M. (2017). Spirulina platensis prevents hyperglycemia in rats by modulating gluconeogenesis and apoptosis via modification of oxidative stress and MAPK-pathways. Biomed. Pharmacother..

[76] Lee S.W., Kim S.M., Hur W., Kang B.-Y., Lee H.L., Nam H., Yoo S.H., Sung P.S., Kwon J.H., Jang J.W. (2021). Tenofovir disoproxil fumarate directly ameliorates liver fibrosis by inducing hepatic stellate cell apoptosis via downregulation of PI3K/Akt/mTOR signaling pathway. PLoS ONE.

[77] Murata K., Tsukuda S., Suizu F., Kimura A., Sugiyama M., Watashi K., Noguchi M., Mizokami M. (2019). Immunomodulatory Mechanism of Acyclic Nucleoside Phosphates in Treatment of Hepatitis B Virus Infection. Hepatology.

[78] Strijdom H., Goswami N., De Boever P., Westcott C., Ogundipe T., Everson F., Genis A. (2016). Cardiometabolic and vascular effects of treatment with a fixed-dose combination anti-retroviral drug containing nucleoside and non-nucleoside reverse transcriptase inhibitors (NRTI s and NNRTI s) in adult rats. Atherosclerosis.

[79] Nsonwu-Anyanwu A.C., Ighodalo E.V., King D., Elochukwu A.C., Jeremiah S., Solomon O.T., Usoro C.A.O. (2017). Biomarkers of Oxidative Stress in HIV Seropositive Individuals on Highly Active Antiretroviral Therapy. React. Oxyg. Species.

[80] Solst S.R., Rodman S.N., Fath M.A., Taylor E.B., Spitz D.R. (2017). Inhibition of Mitochondrial Pyruvate Transport Selectively Sensitizes Cancer Cells to Metabolic Oxidative Stress. Free Radic. Biol. Med..

[81] Tanaka Y., Aleksunes L.M., Yeager R.L., Gyamfi M.A., Esterly N., Guo G.L., Klaassen C.D. (2008). NF-E2-Related Factor 2 Inhibits Lipid Accumulation and Oxidative Stress in Mice Fed a High-Fat Diet. J. Pharmacol. Exp. Ther..

[82] Zhang Z., Zhou S., Jiang X., Wang Y.-H., Li F., Wang Y.-G., Zheng Y., Cai L. (2015). The role of the Nrf2/Keap1 pathway in obesity and metabolic syndrome. Rev. Endocr. Metab. Disord..

[83] de Champlain J., Wu R., Girouard H., Karas M., el Midaoui A., Laplante M.A., Wu L. (2004). Oxidative stress in hypertension. Clin. Exp. Hypertens..

[84] Uruno A., Furusawa Y., Yagishita Y., Fukutomi T., Muramatsu H., Negishi T., Sugawara A., Kensler T.W., Yamamoto M. (2013). The Keap1-Nrf2 System Prevents Onset of Diabetes Mellitus. Mol. Cell. Biol..

[85] Yagishita Y., Fukutomi T., Sugawara A., Kawamura H., Takahashi T., Pi J., Uruno A., Yamamoto M. (2014). Nrf2 Protects Pancreatic-Cells From Oxidative and Nitrosative Stress in Diabetic Model Mice. Diabetes.

[86] Gaikwad A., Long D.J., Stringer J.L., Jaiswal A.K. (2001). In Vivo Role of NAD(P)H:Quinone Oxidoreductase 1 (NQO1) in the Regulation of Intracellular Redox State and Accumulation of Abdominal Adipose Tissue. J. Biol. Chem..

[87] Barbier O., Arreola-Mendoza L., del Razo L.M. (2010). Molecular mechanisms of fluoride toxicity. Chem. Biol. Interact..

